# Enterobiasis: A Neglected or Forgotten Disease?

**DOI:** 10.1590/0037-8682-0323-2024

**Published:** 2024-10-28

**Authors:** Maria Fantinatti, Eduardo José Lopes Torres, Alda Maria Da-Cruz

**Affiliations:** 1 Universidade do Estado do Rio de Janeiro, Faculdade de Ciências Médicas, Disciplina de Parasitologia, Rio de Janeiro, RJ, Brasil.; 2 Instituto Oswaldo Cruz, Fundação Oswaldo Cruz, Laboratório Interdisciplinar de Pesquisas Médicas, Rio de Janeiro, RJ, Brasil.

Intestinal parasitic infections encompass a wide range of diseases caused by intestinal helminths and protozoa that fall under the category of Neglected Tropical Diseases. In April 2023, the federal government of Brazil established the “Interministerial Committee for the Elimination of Tuberculosis and Other Socially Determined Diseases” (In Portuguese, Comitê Interministerial para Eliminação da Tuberculose e Outras Doenças Determinadas Socialmente), which will remain active until 2030. This committee aims to propose intersectoral public policies focused on health equity and the reduction of social inequalities, which are factors directly related to the causes of these diseases. Soil-transmitted helminthiasis (STH) (In Portuguese, geo-helmintíases) are among the “Other Socially Determined Diseases.” Several national surveys and control plans for STH, the most recent in 2018, have been launched in Brazil by the Ministry of Health. Currently, 20 countries endemic for soil-transmitted helminthiasis (Antigua and Barbuda, Argentina, Bolivia, Brazil, Colombia, Cuba, Dominica, El Salvador, Guatemala, Guyana, Haiti, Honduras, Mexico, Nicaragua, Panama, Peru, Saint Vincent and the Grenadines, Paraguay, the Dominican Republic, and Venezuela) participate in meetings to advance key actions to achieve the goal of eliminating by 2030 soil-transmitted helminthiasis as a public health problem in the Americas. The relevance of these public policies is indisputable. However, biohelminths, such as *Enterobius vermicularis* and intestinal protozoa, remain unaddressed, and their prevalence and distributions are unknown. This is particularly concerning when given mass treatment policies aimed at controlling geohelminths while neglecting other enteroparasites, which are often treated with the same drug but not always at the same dosage. This lack of a comprehensive treatment may lead to the selection of resistant parasite strains, owing to the limited availability of a therapeutic arsenal.

Nematodes of the genus *Enterobius* (family Oxyuridae, Linnaeus 1758), which are found in humans and primates, are of anthropozoonotic importance. The relationship between this parasite and its host was first described in the 18^th^ century, although evidence of this relationship dates back to archaeological and paleontological periods. However, several fundamental questions remain regarding the biology, symptomatology, pathogenesis, and epidemiology of the parasite. The knowledge gaps highlighted in our review (Fantinatti and Da-Cruz, 2023) and in a letter by Vitor Luis Tenorio Mati et al.

Underscore the urgent need for investment in basic and applied science in historically neglected intestinal parasitic diseases. A clear example of this gap is the frequently mentioned claim in the medical literature regarding the migration of female parasites to the perianal region at night, despite the lack of scientific evidence and a comprehensive understanding of the biological mechanisms involved. This lack of knowledge contributes to erroneous inferences about important pathophysiological aspects, such as nocturnal perianal itching, which is one of the most prominent symptoms of human enterobiasis. Notably, the significant swimming activity of the adult worm keeps it in the large intestine, because these nematodes lack tissue attachment structures and strategies. This activity is significantly impaired by the accumulation of eggs in the female uterus, a biological reproductive strategy marked by vulvar blockage caused by the cement deposited by the male during copulation, a characteristic observed in other Oxyuridae species that parasitize wild animals^1^. Given these considerations, do female parasites actively migrate to the anal region or are passively transported by peristalsis? Does female migration occur exclusively at night or is this perception influenced by the rest periods of the host? Furthermore, is the infected individual’s awareness heightened at night due to the parasite exclusive activity during this time, or because this period demands the most complete rest for the individual?

The limited investment in basic research on the infectious process and its anatomopathological consequences combined with the absence of dedicated groups for the clinical follow-up of severe or treatment-resistant cases, which are becoming increasingly common, has created a gap that may lead to contentious publications such as reports of fatal cases of human enterobiasis^2^.

Behind the scenes of Brazilian science, we often remark that, in the context of neglected diseases, enterobiasis is one of the “neglected of the neglected.” Ignoring enterobiasis and its impact leads to the normalization of the infection. *E. vermicularis*, commonly known as pinworm, is typically remembered for causing perianal itching. However, it is not uncommon to hear from the population that “worms are a problem for children” or to encounter various unproven home treatments.

Enterobiasis is neglected by healthcare professionals, who often opt for empirical treatment of symptomatic patients, frequently without follow-up. Persistent infection after treatment is observed in the clinical practice but has rarely been reported. Whether this persistence is due to inadequate treatment, reinfection, or parasitic resistance remains unknown. Regardless of the cause, persistent cases lead to prolonged exposure to the parasite-host interaction, which is inevitably harmful; however, the mechanisms involved still need to be elucidated. In addition, the psychosocial impact on infected individuals, especially those with anal itching, must be understood.

Finally, enterobiasis is also neglected by researchers, and a few research groups are investigating this topic. In this context, there is an urgent need for coordinated efforts to promote research on this and other neglected intestinal parasitic diseases. This includes developing national policies and international strategies that go beyond pharmacological approaches, which are often inadequately supported by clinical-scientific evidence, and prioritizing environmental control and monitoring. In conclusion, the current urgency surrounding enterobiasis and other neglected parasitic diseases demands strategic alignment with the Sustainable Development Goals (SDG) of the 2030 Agenda adopted by United Nations members. This approach should promote an integrated strategy that strengthens basic science and innovation to achieve sustainable and effective solutions for controlling these diseases, particularly emphasizing SDG 3 (Good Health and Well-being) and SDG 4 (Quality Education).

We conclude by reiterating the final questions from the letter by Vitor Luis Tenorio Mati et al.: “How can we motivate policymakers, institutions, and researchers to overcome inertia and actively pursue studies related to enterobiasis?” and “What strategies can be implemented to enhance research efforts in this field, encompassing funding and grants, data sharing, repositories, and international cooperation?”
